# Temporal Changes in Brain Light Scattering and Its Independent Variables Within 2 Days of Life

**DOI:** 10.3390/bios15120818

**Published:** 2025-12-17

**Authors:** Kennosuke Tsuda, Sachiko Iwata, Shinji Saitoh, Osuke Iwata

**Affiliations:** Center for Human Development and Family Science, Department of Pediatrics and Neonatology, Nagoya City University Graduate School of Medical Sciences, Nagoya 467-8601, Japano.iwata@med.nagoya-cu.ac.jp (O.I.)

**Keywords:** time-resolved near-infrared spectroscopy, reduced scattering coefficient, newborn infants, cerebral microstructure

## Abstract

The reduced scattering coefficient (μ_s_′), measured using time-resolved near-infrared spectroscopy (TR-NIRS) has been linked to brain water diffusion assessed by diffusion tensor imaging, suggesting its potential as a bedside marker of cerebral microstructure. However, the physiological determinants of μ_s_′ and its early postnatal changes remain unclear. This study examined clinical associations with cerebral μ_s_′ in healthy term newborn infants during the first 2 postnatal days. Eighteen newborn infants underwent TR-NIRS at 6 and 36 h postnatally. Associations between μ_s_′ and 14 clinical variables were analysed using generalised estimating equations. Median μ_s_′ was 7.395 cm^−1^ (IQR: 6.140–8.159) at 6 h and 7.112 cm^−1^ (IQR: 6.473–7.410) at 36 h, with no significant difference (*p* = 0.327). Male sex was associated with higher μ_s_′ (regression coefficient = 0.895, *p* = 0.007), whereas caesarean delivery (regression coefficient = −0.969, *p* = 0.012) was associated with lower μ_s_′. A significant interaction between caesarean delivery and postnatal age indicated that the negative effect diminished between 6 and 36 h after birth (difference = 0.057, *p* = 0.016). These findings suggest delivery mode transiently influences brain scattering, whereas the effect of sex remains stable, supporting further investigation of TR-NIRS as an acute-phase cerebral marker.

## 1. Introduction

Perinatal brain injury affects 1–3 per 1000 live births in developed countries [1,2], presenting a major challenge in neonatal care. Among survivors, 33–50% develop severe neurodevelopmental disabilities, including cerebral palsy and intellectual disability [3]. Early and accurate assessment of cerebral physiology is crucial for identifying newborn infants at risk, guiding neuroprotective strategies and improving outcomes [4]. The hyperacute phase—within the first hours to days after birth—is particularly critical, characterised by a rapid and evolving cascade involving impaired oxidative metabolism, activation of anaerobic pathways, depletion of adenosine triphosphate, excitotoxic neurotransmitter release, reperfusion injury and cytotoxic brain oedema [5,6,7,8,9]. At the bedside, clinicians often infer the progression of this cascade from neurological examination and brain function monitors such as electroencephalography [10,11,12]; however, the predictive value of these conventional tools is limited [13,14]. Novel approaches capable of capturing early cerebral structural and functional alterations may therefore improve diagnostic precision and facilitate timely neuroprotective intervention.

Magnetic resonance imaging (MRI) and magnetic resonance spectroscopy (MRS) provide robust biomarkers, with reduced apparent diffusion coefficient [15,16] and elevated lactate-to-N-acetylaspartate ratios (24–96 h post-insult) [17,18] strongly predicting neurological outcomes. However, both of them are rarely feasible in the hyperacute phase because of safety and logistical challenges. Imaging is typically undertaken only after stabilisation in intensive care, limiting the ability to monitor dynamic cerebral changes, including cytotoxic oedema [8] and microstructural disruption of cytoplasmic organelles [19], occurring between the initial insult and delayed imaging. Furthermore, MRI requires transfer to dedicated facilities, often with the use of sedative drugs, and is impractical for unstable or preterm infants. These constraints have catalysed interest in optical techniques capable of providing complementary, real-time information on cerebral metabolism and structure directly at the bedside.

Near-infrared spectroscopy (NIRS) is a non-invasive, bedside technique widely used to assess cerebral oxygenation in infants and neonates [20]. Conventional continuous-wave NIRS provides information regarding the fraction of oxygenated and deoxygenated haemoglobin by detecting wavelength-dependent differences in light absorption and applying known extinction coefficients [21]. Although valuable for clinical monitoring, continuous-wave NIRS fundamentally measures changes in light attenuation and cannot independently distinguish between tissue absorption and scattering. This limits its depth sensitivity and prevents absolute quantification [22]. Importantly, the brain is composed not only of vasculature, but also of neurons, glial cells and other parenchymal structures that influence light absorption and scattering [23]. Therefore, separating these optical effects is essential for accurately interpreting physiological and microscopic structural changes during neonatal brain development. Light scattering is affected by cellular morphology, membrane structure and water distribution [24,25], and scattering-sensitive measurements may provide important information about tissue microstructure that conventional NIRS cannot reveal.

Time-resolved NIRS (TR-NIRS) addresses these limitations by utilising ultrashort pulses of near-infrared light and recording the temporal distribution of photon arrival times after transcranial passage [26,27]. Analysis of photon arrival profiles allows for the independent determination of the absorption coefficient (μ_a_), which reflects chromophore concentrations and tissue oxygenation, and the reduced scattering coefficient (μ_s_′), which reflects microstructural tissue characteristics such as cellular density and water content [24,25]. Measurements of μ_s_′ have been shown to correlate with macrostructural injury scores and diffusion tensor imaging parameters [28]. Recent technical advances have improved temporal resolution, quantification accuracy and signal-to-noise ratio in TR-NIRS [29], increasing its potential to detect subtle cerebral changes associated with maturation or injury. Several studies have demonstrated feasibility in term and preterm newborn infants, indicating that TR-NIRS can be safely implemented even in the immediate postnatal period [28,30,31,32,33,34,35].

It is expected that μ_s_′ may be sensitive to early microstructural alterations and could substitute for MRI. However, to realise this potential, it is first necessary to establish the physiological variation of μ_s_′ and its temporal trajectory after birth in healthy newborn infants. During the transition to extrauterine life, newborn infants experience substantial physiological adaptations, including increased oxygen concentration, systemic haemodynamic shifts and contraction of extracellular volume [36,37]. The impact of these postnatal changes on μ_s_′ remains poorly understood. Moreover, factors such as delivery mode, sex and perinatal stress may transiently influence cerebral water distribution and microstructural organisation, highlighting the need to disentangle normal developmental trajectories from pathological signals [38,39,40]. Establishing reference trends in μ_s_′ could therefore provide a foundation for interpreting deviations associated with hypoxia–ischaemia or other acute encephalopathies. Establishing a normative baseline for scattering behaviour is therefore essential for distinguishing pathological changes and enhancing the early diagnosis and treatment of newborn infants at risk of perinatal brain injury.

In this study, we aimed to examine the dependence of μ_s_′ values on key clinical variables, including delivery mode and sex, and to characterise their temporal evolution after birth using TR-NIRS. By defining the normal postnatal behaviour of scattering properties during the first days of life, this work seeks to provide fundamental insight into neonatal brain microstructural adaptation and to support future clinical application of TR-NIRS in the early detection of cerebral injury.

## 2. Materials and Methods

This prospective observational study was conducted in accordance with the Declaration of Helsinki. The protocol was approved by the ethics committee of Nagoya City University (reference number: 60-22-0031). Families received verbal and written explanations of the study, and informed consent was obtained.

### 2.1. Study Population

Nineteen healthy newborn infants with gestational age ≥36 weeks were initially screened in the labour ward of Nagoya City University Hospital between May and December 2023. Only clinically well newborn infants were included; those requiring neonatal intensive care unit admission were excluded. One infant was excluded because parental consent could not be obtained, leaving a final cohort of 18 newborn infants for analysis.

### 2.2. TR-NIRS Measurement

Data collection was performed at approximately 6 and 36 h after birth, with newborn infants measured at rest. The 6 h time point was selected because it lies within the therapeutic window relevant for clinical decision-making in hypoxic–ischaemic encephalopathy [41]. The 36 h time point was selected because early neonatal physiological adaptation is largely complete by this stage [42].

Measurements were obtained using TR-NIRS (TRS-21, Hamamatsu Photonics K.K., Hamamatsu, Shizuoka, Japan). The TR-NIRS probe, inserted in a rubber holder (inter-optode distance 3 cm), was applied to a flat cranial area so that the source and detector surfaces were aligned. A time-correlated single-photon counting method measured μ_s_′ values at 765, 795 and 830 nm in the frontal region [28,34,35]. In addition to scattering, absorption-related variables, including tissue oxygen saturation (ScO_2_), total haemoglobin concentration (total Hb) and μ_a_, were obtained concurrently to provide complementary physiological context, although variables were not the primary focus of the present analysis. Each 10 s acquisition was repeated five times with probe repositioning; readings were averaged. Data were inspected retrospectively for reproducibility and fit to the photon diffusion equation. Replicates with unreliable photon-fitting, typically resulting from motion or signal instability, were automatically flagged and excluded by the device’s built-in algorithm. Overall, 7% of replicates were excluded using this procedure, and the remaining data were included in the analyses.

### 2.3. Clinical Information

Clinical information was obtained from patient records: (i) maternal and antenatal factors (parity, mode of delivery, preterm rupture of membranes); (ii) birth and admission data (sex, gestational age, body weight, head circumference, Apgar score at 5 min, cord blood gases, need for resuscitation); and (iii) study-time variables (postnatal age) (Table 1). Japanese standards were used to calculate z-scores of birth weight and head circumference [43].

### 2.4. Data Analysis

Data are presented as mean ± standard deviation (SD) for normally distributed variables and median [interquartile range (IQR)] for non-normal distributions. Because scattering properties were consistent across wavelengths, μ_s_′ values at 765 nm were used for analysis [34]. To assess repeatability, intraclass correlation coefficient (ICC) values were calculated. A two-way mixed-effects model with absolute agreement was applied. ICC values were reported for single and average measures, together with 95% confidence interval (95% CI). Following established guidelines, ICC values < 0.50 were considered poor; 0.50–0.75, moderate; 0.75–0.90, good; and >0.90, excellent reliability [44]. In addition, Levene’s test was performed to evaluate the homogeneity of variance in μ_s_′ values between different postnatal ages.

Generalised estimating equations assessed intra-individual changes and evaluated interactions between μ_s_′ values and postnatal age, with participant IDs accounting for repeated measures (SPSS v29, IBM, Armonk, NY, USA). Univariable linear regression explored 14 clinical variables, selected for biological relevance and prior evidence, focusing on intrauterine growth and perinatal distress (Table 2). For exploratory analyses, *p*-values were not corrected for multiple comparisons. Interactions with postnatal age were tested for continuous independent variables; categorical interactions were assessed only when contingency table cell counts exceeded three because statistical power was limited.

## 3. Results

### 3.1. Baseline Characteristics

In the study cohort, caesarean section was performed in eight of 18 cases (44.4%). In addition, three cases (16.7%) were classified as emergency deliveries, comprising emergency caesarean, forceps and vacuum-assisted vaginal births. Premature rupture of membranes occurred in three cases (16.7%). The mean gestational age was 38.7 ± 1.1 weeks, and the mean birth weight was 2817 ± 335 g. Median Apgar score at 5 min was 9 [9–9]. Two newborn infants (11.1%) required resuscitation at birth; both received mask-based continuous positive airway pressure for 7 and 3 min, respectively, and no further respiratory support was required thereafter (Table 1).

### 3.2. Longitudinal Changes in Reduced Scattering Coefficients

**Figure 1:**
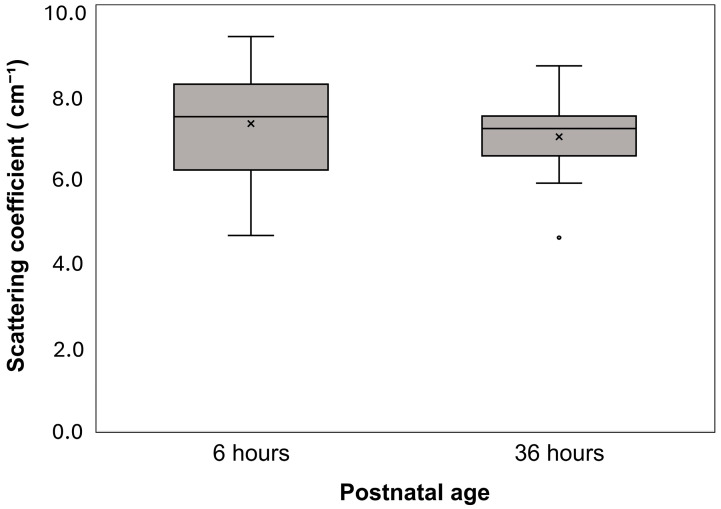
Postnatal changes in reduced scattering coefficient (μ_s_′). Boxplots show the distribution of μ_s_′ values for all infants at 6 and 36 h after birth. Boxes represent the first and third quartiles; the horizontal line inside each box indicates the median; whiskers extend to the most extreme values within 1.5 times the interquartile range; × denotes the mean; and open circles indicate outliers beyond 1.5 times the interquartile range.

Median μ_s_′ values were 7.395 cm^−1^ [6.140–8.159] at 6 h and 7.112 cm^−1^ [6.473–7.410] at 36 h, with no statistically significant difference between the two time points (*p* = 0.327). Levene’s test showed no significant difference in variance (*p* = 0.095), supporting the assumption of homogeneity of variance (Figure 1).

### 3.3. Repeatability Analysis

The ICC (two-way mixed, absolute agreement) was 0.94 (95% CI: 0.89–0.97) for single measures, indicating that even a single acquisition provided highly reliable values. When five repeated measurements were averaged, the ICC increased to 0.99 (95% CI: 0.98–1.00), demonstrating excellent reliability of the averaged values used for subsequent analyses.

### 3.4. Absorption-Related Parameters

Median ScO_2_ was 64.9% [63.5–67.1] at 6 h and increased to 70.6% [67.1–72.5] at 36 h, showing a statistically significant difference between time points (*p* = 0.010). Total Hb was 61.9 µM [53.3–71.7] at 6 h and 60.6 µM [54.4–71.3] at 36 h, with no significant difference observed (*p* = 0.586). Similarly, μ_a_ remained stable, with values of 0.160 cm^−1^ [0.146–0.176] at 6 h and 0.156 cm^−1^ [0.142–0.173] at 36 h (*p* = 0.472). Boxplots depicting these results are provided in Figures S1–S3.

### 3.5. Crude Associations Between Clinical Backgrounds and μ_s_′ Values

**Figure 2:**
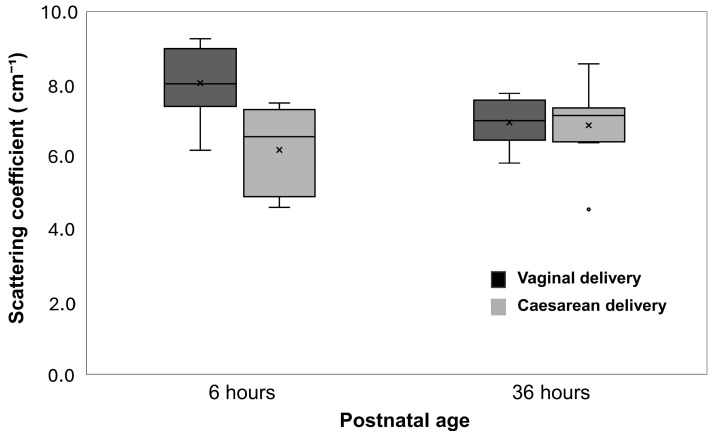
Changes in the reduced scattering coefficient (μ_s_′) by mode of delivery. Boxplots illustrate the μ_s_′ values at 6 and 36 h after birth in newborn infants delivered by caesarean section and vaginal delivery. Boxes represent the first and third quartiles; the horizontal line inside each box indicates the median; whiskers extend to the most extreme values within 1.5 times the interquartile range; × denotes the mean; and open circles indicate outliers beyond 1.5 times the interquartile range.

**Figure 3:**
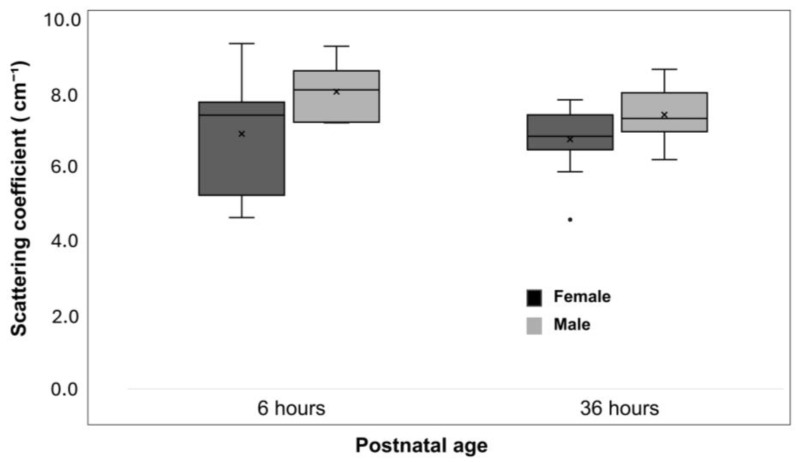
Changes in the reduced scattering coefficient (μ_s_′) by sex. Boxplots show the μ_s_′ values at 6 and 36 h after birth in male and female infants. Boxes represent the first and third quartiles; the horizontal line inside each box indicates the median; whiskers extend to the most extreme values within 1.5 times the interquartile range; × denotes the mean; and open circles indicate outliers beyond 1.5 times the interquartile range.

Newborn infants delivered by caesarean section had lower μ_s_′ values than those delivered vaginally (regression coefficient, –0.969; 95% CI, –1.726 to –0.211; *p* = 0.012) (Table 2 and Figure 2). Male newborn infants tended to have higher μ_s_′ values compared with females (regression coefficient, 0.895; 95% CI, 0.248–1.543; *p* = 0.007) (Table 2 and Figure 3).

### 3.6. Influence of Postnatal Age on the Associations Between Clinical Backgrounds and μ_s_′ Values

Caesarean delivery showed a significant interaction with postnatal age, with a significantly increased regression coefficient between μ_s_′ values and postnatal age (difference in regression coefficient, 0.057; 95% CI, 0.011–0.103; *p* = 0.016) (Table 3 and Figure 2). In contrast, male sex did not demonstrate a significant interaction with postnatal age (*p* = 0.802) (Table 3 and Figure 3).

## 4. Discussion

In healthy term newborn infants, we observed a non-significant but consistent reduction in μ_s_′ between 6 and 36 h after birth. Previous studies have reported that μ_s_′ during the first week of life increases with gestational age [45]. Our findings suggest that μ_s_′ undergoes short-term fluctuations linked to birth transition and that its association with clinical variables evolves with postnatal age. To establish μ_s_′ as a reliable bedside biomarker for critically ill newborn infants, it is essential to characterise these temporal changes in larger cohorts of healthy term newborn infants.

The optical scattering of near-infrared light within biological tissue theoretically increases with greater microstructural complexity, as multiple scattering events extend photon path length and reflection [26]. Thus, the μ_s_′ metric has the potential to serve as a surrogate marker for tissue microstructure. In newborn infants with illness, μ_s_′ within the first week has been reported to correlate strongly with gestational age [45], Apgar score and umbilical cord pH [34]. It also correlates with structural and microstructural MRI parameters near term-equivalent age [28]. However, physiological transitions during the early postnatal period may also influence μ_s_′. For example, a 5–10% reduction in interstitial fluid content occurs within the first few days after birth [37], potentially coinciding with spatial changes in water distribution within the brain tissue [46]. Understanding the postnatal trajectories of μ_s_′ in healthy newborn infants, as well as its dependence on clinical variables at each stage, is therefore critical.

In the present study, delivery mode and sex were identified as clinical factors associated with μ_s_′ immediately after birth, although these differences diminished by 36 h of age. The transient nature of these associations suggests that the determinants of μ_s_′ are likely time specific, reflecting the dynamic physiological adjustments that characterise the early neonatal period.

Regarding the delivery mode, it is well established that newborn infants delivered by elective caesarean section do not experience characteristic surges in stress-related hormones, most notably catecholamines and cortisol, that normally occur during labour [47,48]. These hormonal changes could influence neonatal fluid regulation [49] and cerebral microstructure [50], supporting the observed differences in μ_s_′ between different delivery modes. In addition, previous studies have shown that delivery mode may affect systemic hemodynamic adaptation during the early transitional period. Specifically, elective caesarean delivery has been associated with the timing of ductus arteriosus closure [51] and differences in cerebral blood volume, suggestive of altered redistribution of cardiac output to the cerebral circulation [31]. These haemodynamic factors may interact with the hormonal milieu to modify cerebral optical properties. In our study, however, an interaction between caesarean delivery and postnatal age was observed, demonstrating that the negative effect of Caesarean delivery to μ_s_′ values diminished over time, suggesting that the influence of delivery mode on brain scattering is only transient. Although these mechanisms provide a biologically plausible framework, their contributions remain speculative in the absence of concurrent measurements of hormonal profiles, cardiac output and ductal shunting in this cohort. Further studies integrating these physiological variables are needed to clarify the pathways through which delivery mode influences early postnatal cerebral light scattering.

Sex-related differences in μ_s_′ may similarly reflect underlying variations in neonatal brain structure. Large-scale MRI studies have shown that male newborn infants typically exhibit larger head circumference and total brain volume than females, even after adjustment for body size [38,52]. Although no significant sex difference in head circumference was observed in our cohort, subtler divergences in structural organisation, such as differences in the white and grey matter ratio, microstructural compactness and water content, may still exist. In addition, early postnatal hormonal influences, including transient surges in testosterone and oestradiol [53], could contribute to differential maturation patterns that affect scattering properties. These factors may partly explain the modest associations between the sex and μ_s_′ values. Future studies with larger cohorts and complementary imaging approaches could help clarify the biological basis of these sex-related differences in values.

Even within the short span of 36 h after birth, substantial physiological and structural adaptations occur, including the initiation of spontaneous breathing [54], the transition from foetal to neonatal circulation [55] and redistribution of body water compartments. These processes likely drive concurrent changes in cerebral microstructure that are detectable by TR-NIRS. This highlights a unique strength of TR-NIRS as a tool for monitoring early postnatal brain adaptation, enabling repeated, non-invasive, bedside assessment of microstructural dynamics during this critical transition period. In TR-NIRS neuromonitoring, short pulses of light are delivered into the head, and the arrival time of each photon at the detector is precisely measured to generate a distribution of time-of-flight. This allows differentiation of early-arriving photons, which have only passed through extracerebral layers (scalp, skull and cerebrospinal fluid), from late-arriving photons, which are more likely to have travelled through the complex part of the brain [56]. In the longer term, longitudinal TR-NIRS profiling may help define normative patterns of brain microstructural maturation and assist in the early identification of newborn infants at risk for neurological complications or suboptimal neurodevelopmental trajectories.

This study has some limitations. First, mainly because of the limited time interval from birth to the recruitment of newborn infants and the first data acquisition within 6 h of birth, we were able to accumulate only a small sample size, rendering most statistical comparisons susceptible to both type-1 and type-2 errors. Similarly, we were unable to account for several important covariates. For example, all infants scored Apgar scores of 9 except for one who scored 8, where statistical analysis was not performed. In addition, there was a trend that the inter-individual deviation of μ_s_′ became smaller from 6 to 36 h after birth, which did not reach the statistical significance. Future studies in larger cohorts need to clarify whether dynamic physiological transitions after birth transiently increase the inter-individual variability of μ_s_′ values even in healthy newborn infants. Despite the small study population, measurement repeatability was excellent; the ICC was 0.94 for single measures and 0.99 for averaged measures, indicating that the μ_s_′ values used in the analyses were highly reliable. Additionally, although full adjustment for potential confounders was not possible, by using generalised estimating equations, repeated within-subject measures were incorporated [57], leading to successful detection of overall temporal trends. Second, unlike our previous studies in hospitalised infants, all measurements in the current study were obtained from the frontal region, despite that microstructural changes after birth may not be uniform across different brain regions [58]. In addition, although we tried to apply the TR-NIRS probe vertically to the relatively flat part of the forehead, it is still possible that the measurement was affected by the subtle curve of the scalp, because the estimation of light scattering relies on the diffusion theory, which assumes a flat semi-infinite medium [59].

## 5. Conclusions

In healthy term newborn infants, μ_s_′ showed a non-significant but consistent decrease between 6 and 36 h after birth. Together with previous evidence that μ_s_′ varies with maturity [45], our findings suggest that μ_s_′ is sensitive to the rapid physiological changes accompanying the transition from intrauterine to extrauterine life. Rather than remaining static, μ_s_′ undergoes early temporal changes that likely reflect evolving cerebral microstructure, water content and haemodynamic adaptation. The influence of clinical factors on μ_s_′, including delivery mode and sex, was greatest immediately after birth and diminished by 36 h after birth, indicating that the determinants of μ_s_′ are closely linked to early postnatal physiological adjustments. Thus, interpretation of μ_s_′ requires careful consideration of postnatal age and perinatal conditions.

Our findings also support the potential of TR-NIRS as a bedside tool for capturing these rapid microstructural adaptations. Longitudinal profiling may help define normative trajectories of cerebral maturation and identify deviations associated with illness or suboptimal neurodevelopment.

Further longitudinal studies in larger cohorts of healthy newborn infants are needed to refine normative μ_s_′ ranges and delineate its temporal evolution across the early neonatal period. Integration with complementary measures, such as cerebral blood flow, perfusion indices and tissue water content, will strengthen the physiological interpretation of μ_s_′ and advance its translation into clinical neurocritical care.

## Figures and Tables

**Table 1:** Clinical background variables of the study population.

Variables	Values
Maternal and antenatal variables	
Nulliparity	10 (55.6%)
Multiple pregnancy	1 (5.6%)
Premature rupture of membranes	3 (16.7%)
Caesarean delivery	8 (44.4%)
Emergency delivery	3 (16.7%)
Variables at birth	
Male sex	6 (33.3%)
Gestational age (weeks)	38.7 (1.1)
Body weight (g)	2817 (335)
Head circumference (cm)	33.3 (1.2)
Intrauterine growth restriction	1 (5.6%)
Apgar score at 5 min	9 [9–9]
Cord blood analysis	
pH	7.31 (0.05)
Base excess (mmol/L)	−3.5 (1.6)
Need for resuscitation	2 (11.1%)
Variables at the time of study	
Postnatal age at the time of 1st TR-NIRS study (h)	5.0 (1.8)
Postnatal age at the time of 2nd TR-NIRS study (h)	30.5 (4.6)

Values are presented as numbers (%), means (standard deviation), or medians [interquartile range]. Abbreviation: TR-NIRS, time-resolved near-infrared spectroscopy.

**Table 2:** Independent variable associated with the reduced scattering coefficient.

Variables	Regression Coefficient	95% CI		*p*
		Lower	Upper	
Maternal and antenatal variables				
Nulliparity	0.133	−0.694	0.959	0.753
Premature rupture of membranes	0.548	−0.339	1.435	0.226
Caesarean delivery	−0.969	−1.726	−0.211	0.012
Emergency delivery	0.333	−0.706	1.373	0.530
Variables at birth				
Male sex	0.895	0.248	1.543	0.007
Gestational age (weeks)	0.318	−0.183	0.819	0.214
Body weight (g)	0.001	0.000	0.003	0.082
Body weight z-score	0.242	−0.197	0.681	0.281
Head circumference (cm)	−0.015	−0.301	0.271	0.920
Head circumference z-score	−0.083	−0.449	0.282	0.655
Cord blood analysis				
pH	−3.722	−7.924	0.479	0.082
Base excess (mmol/L)	0.246	−0.063	0.555	0.119
Need for resuscitation	0.405	−0.289	1.099	0.253
Variables at the time of study				
Postnatal age (h)	−0.021	−0.046	0.004	0.093

Abbreviation: CI, confidence interval.

**Table 3:** Influence of postnatal age on the relationship between clinical characteristics and the reduced scattering coefficient.

Variables	Regression Coefficient	95% CI		*p*
		Lower	Upper	
Caesarean delivery	−0.046	−0.070	−0.023	<0.001
Postnatal age (h)	−1.943	−2.975	−0.911	<0.001
Caesarean delivery × Postnatal age	0.057	0.011	0.103	0.016
Male sex	0.980	−0.140	2.100	0.086
Postnatal age (h)	−0.018	−0.049	0.013	0.251
Male sex × Postnatal age	−0.006	−0.054	0.042	0.802
Gestational age (weeks)	0.367	−0.325	1.059	0.298
Postnatal age (h)	0.113	−0.585	0.811	0.752
Gestational age × Postnatal age	−0.003	−0.021	0.015	0.709
Birth weight z-score	0.317	−0.677	1.310	0.532
Postnatal age (h)	−0.022	−0.053	0.008	0.532
Birth weight z-score × Postnatal age	−0.004	−0.040	0.031	0.819

× indicates an interaction term between the listed variable and postnatal age. Abbreviation: CI, confidence interval.

## Data Availability

The data presented in this study are available on request from the corresponding author.

## Supplementary Materials

The following supporting information can be downloaded at: https://www.mdpi.com/article/10.3390/bios15120818/s1, Figure S1: Postnatal changes in tissue oxygenation saturation (ScO_2_). Boxplots show the distribution of ScO_2_ values for all infants at 6 and 36 h after birth. Boxes represent the first and third quartiles; the horizontal line inside each box indicates the median; whiskers extend to the most extreme values within 1.5 times the interquartile range; and × denotes the mean; Figure S2: Postnatal changes in total haemoglobin concentration (total Hb). Boxplots show the distribution of total Hb values for all infants at 6 and 36 h after birth. Boxes represent the first and third quartiles; the horizontal line inside each box indicates the median; whiskers extend to the most extreme values within 1.5 times the interquartile range; and × denotes the mean; Figure S3: Postnatal changes in absorption coefficient (μ_a_). Boxplots show the distribution of μ_a_ values for all infants at 6 and 36 h after birth. Boxes represent the first and third quartiles; the horizontal line inside each box indicates the median; whiskers extend to the most extreme values within 1.5 times the interquartile range; and × denotes the mean.

## Abbreviations

The following abbreviations are used in this manuscript:

CI	Confidence Interval
ICC	Intraclass Correlation Coefficient
IQR	Interquartile Range
MRI	Magnetic Resonance Imaging
MRS	Magnetic Resonance Spectroscopy
NIRS	Near-Infrared Spectroscopy
ScO_2_	Tissue Oxygen Saturation
SD	Standard Deviation
SPSS	Statistical Package for the Social Sciences
Total Hb	Total Haemoglobin Concentration
TR-NIRS	Time-Resolved Near-Infrared Spectroscopy
μ_s_′	Reduced Scattering Coefficient
μ_a_	Absorption Coefficient
