# Supplementary material for: Temporal Changes in Brain Light Scattering and Its Independent Variables Within 2 Days of Life
Source: Biosensors (Basel). 2025 Dec 17;15(12):818. doi: 10.3390/bios15120818 (PMC12730957; doi:10.3390/bios15120818)
Supplement: Supplementary file 1 [file biosensors-15-00818-s001.zip › biosensors-3940458-supplementary.pdf]

Supplementary Materials

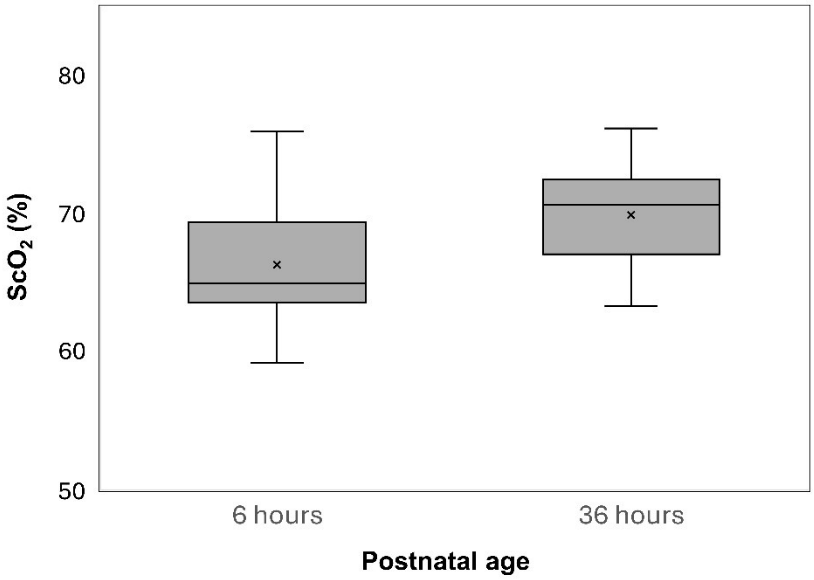

Figure S1. Postnatal changes in tissue oxygenation saturation (ScO_2). Boxplots show the distribution of ScO_2 values for all infants at 6 and 36 h after birth. Boxes represent the first and third quartiles; the horizontal line inside each box indicates the median; whiskers extend to the most extreme values within 1.5 times the interquartile range; and × denotes the mean.

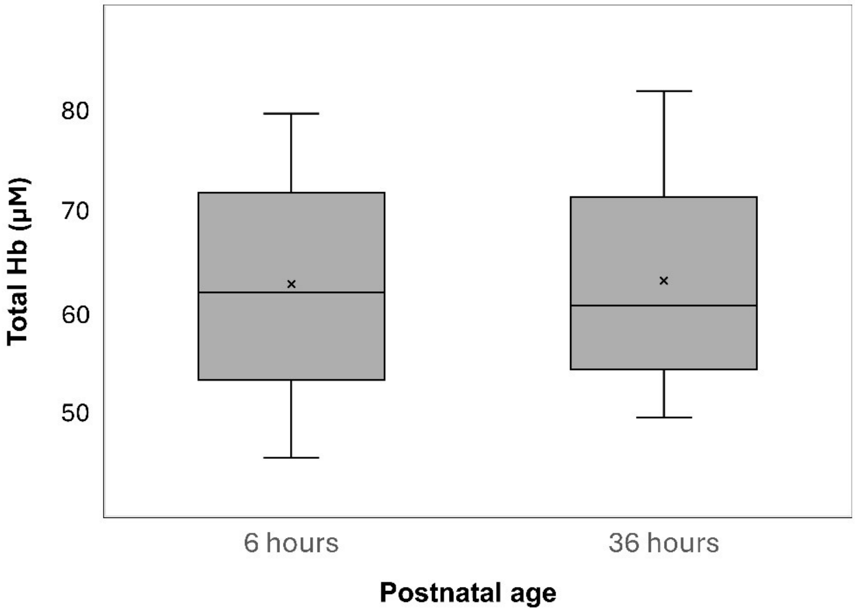

Figure S2. Postnatal changes in total haemoglobin concentration (total Hb). Boxplots show the distribution of total Hb values for all infants at 6 and 36 h after birth. Boxes represent the first and third quartiles; the horizontal line inside each box indicates the median; whiskers extend to the most extreme values within 1.5 times the interquartile range; and × denotes the mean.

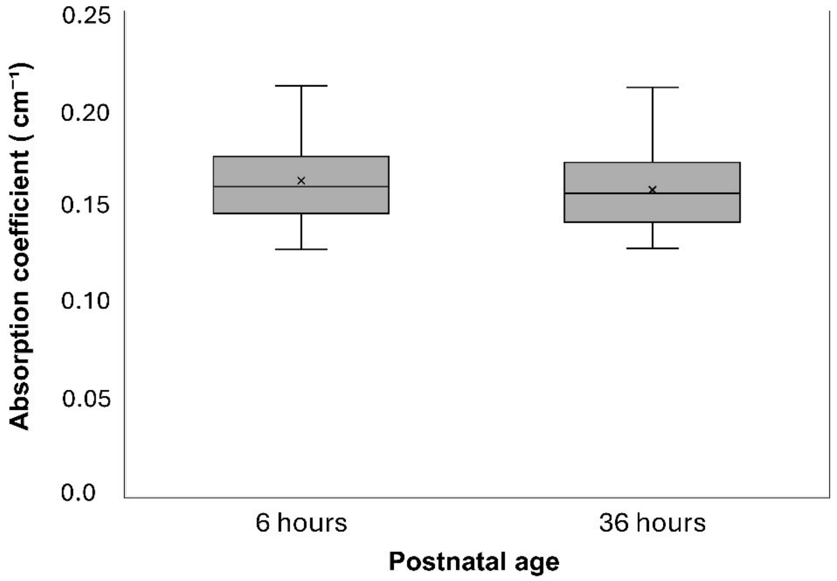

Figure S3. Postnatal changes in absorption coefficient (μ_a). Boxplots show the distribution of μ_a values for all infants at 6 and 36 h after birth. Boxes represent the first and third quartiles; the horizontal line inside each box indicates the median; whiskers extend to the most extreme values within 1.5 times the interquartile range; and × denotes the mean.
